# Phosphatidylcholine (18:0/20:4), a potential biomarker to predict ethionamide‐induced hepatic steatosis in rats

**DOI:** 10.1002/jat.4324

**Published:** 2022-03-29

**Authors:** Kyotaka Muta, Kosuke Saito, Yusuke Kemmochi, Taku Masuyama, Akio Kobayashi, Yoshiro Saito, Shoichiro Sugai

**Affiliations:** ^1^ Toxicology Research Laboratories, Central Pharmaceutical Research Institute Japan Tobacco Inc. Yokohama Japan; ^2^ Division of Medicinal Safety Science National Institute of Health Sciences Kawasaki Japan

**Keywords:** DILI, lipid profile, lipidomics, triglyceride, very low‐density lipoprotein

## Abstract

Ethionamide (ETH), a second‐line drug for multidrug‐resistant tuberculosis, is known to cause hepatic steatosis in rats and humans. To investigate predictive biomarkers for ETH‐induced steatosis, we performed lipidomics analysis using plasma and liver samples collected from rats treated orally with ETH at 30 and 100 mg/kg for 14 days. The ETH‐treated rats developed hepatic steatosis with Oil Red O staining‐positive vacuolation in the centrilobular hepatocytes accompanied by increased hepatic contents of triglycerides (TG) and decreased plasma TG and total cholesterol levels. A multivariate analysis for lipid profiles revealed differences in each of the 35 lipid species in the plasma and liver between the control and the ETH‐treated rats. Of those lipids, phosphatidylcholine (PC) (18:0/20:4) decreased dose‐dependently in both the plasma and liver. Moreover, serum TG‐rich very low‐density lipoprotein (VLDL) levels, especially the large particle fraction of VLDL composed of PC containing arachidonic acid (20:4) involved in hepatic secretion of TG, were decreased dose‐dependently. In conclusion, the decreased PC (18:0/20:4) in the liver, possibly leading to suppression of hepatic TG secretion, was considered to be involved in the pathogenesis of the ETH‐induced hepatic steatosis. Therefore, plasma PC (18:0/20:4) levels are proposed as mechanism‐related biomarkers for ETH‐induced hepatic steatosis.

## Abstract 2

Lipidomics analysis was performed in the plasma and liver of ETH‐treated rats to investigate biomarkers for ETH‐induced hepatic steatosis. The ETH‐treated rats showed a dose‐dependent decrease in PC (18:0/20:4) in the plasma and liver and hepatic steatosis with a decrease in serum TG‐rich VLDL levels. In conclusion, plasma PC (18:0/20:4) levels are proposed as mechanism‐related biomarkers for ETH‐induced hepatic steatosis.

## 1 INTRODUCTION

Drug‐induced liver injury (DILI) is a serious concern for subjects and patients who are administered drugs and also for pharmaceutical companies. DILI often leads to discontinuation of the clinical development or withdrawal from the market (Parasrampuria et al., 2018); hence, prediction and risk management of DILI is an urgent issue for pharmaceutical companies (Kullak‐Ublick et al., 2017). Some drugs causing DILI are known to induce haptic steatosis in laboratory animals and humans (Le et al., 1988; Yann et al., 1994), and hepatic steatosis is regarded as one of components of DILI (Bessone et al., 2018; Pavlik et al., 2019). Therefore, to clarify the mechanism, and find predictive biomarkers, of hepatic steatosis is important in the prediction and risk management of DILI.

Ethionamide (ETH) is prescribed as a second‐line drug for multidrug‐resistant tuberculosis (Tiberi et al., 2019; Tousif et al., 2015) and has chemical properties as a prodrug activated by the flavin‐dependent monooxygenase enzyme, *ethA*, of *Mycobacterium tuberculosis* (Nikiforov et al., 2016; Wang et al., 2007). ETH is known to often cause DILI characterized by hepatic necrosis and jaundice in humans (Hollinrake, 1968; Lees, 1963; Moulding & Goldstein, 1962; Phillips & Tashman, 1963). ETH is reported to cause hepatic steatosis with accumulation of lipid droplets in rats (Sahini et al., 2014) followed by hepatic injury associated with endoplasmic reticulum (ER) stress (Sutherland et al., 2018). However, the pathogenesis of hepatic steatosis or hepatic injury by ETH is not fully understood.

Although the lipid droplets in hepatic steatosis mainly consist of triglycerides (TG), the elucidation of the mechanisms is not so easy due to diverse cellular events related to hepatic steatosis (Schumacher & Guo, 2015). Lipidomics, a novel analytical method for lipid expression profiling, enables us to perform detailed lipid profiling (Dehairs et al., 2015; Han & Gross, 2005; Houjou et al., 2005; Triebl et al., 2017; Vihervaara et al., 2014). This analytical technology is effectively utilized in many in vitro and in vivo experiments for clarifying lipid profiles and substantially contributes not only to elucidate the mechanisms of the diseases but also to the discovery of biomarkers for diseases and drug‐induced injuries (Dehairs et al., 2015; Saito, 2021; Triebl et al., 2017). Because lipids work as a component of the lipid bilayer, as well as for the storage of energy and as intracellular and extracellular signal transducers (Simons & Toomre, 2000; Welte & Gould, 2017), alteration in the lipid profile is considered to be involved as a step in diverse diseases. In fact, some studies using lipidomics have revealed that the lipid profile is altered in the onset of cancer and Alzheimer's disease (Kao et al., 2020; Saito et al., 2016). Lipidomics has been also applied for some evaluations of drug‐induced hepatic steatosis. Goda et al. (2018) demonstrated that hepatic steatosis model rats treated with valproic acid showed decreases in plasma ether‐phosphatidylcholines (ePCs) including PC (16:0e/22:4) and PC (16e:22:6) via downregulation of the *glycerone‐phosphate O‐acyltransferase* (*Gnpat*) gene synthesizing ePCs in peroxisomes. This result indicated that changes in plasma lipids were available for exploring as biomarkers for drug‐induced hepatic steatosis (DIS).

In the present study, we conducted lipidomics analysis for the plasma and liver lipids in an ETH‐induced hepatic steatosis model rat, in which hepatic necrosis was avoided by using lower doses, to discover predictive biomarkers for ETH‐induced hepatic steatosis.

## 2 MATERIALS AND METHGODS

### 2.1 Animals

Male Sprague–Dawley (Crl:CD [SD]) rats (5 weeks old) were purchased from Charles River Japan Inc. (Kanagawa, Japan) and were quarantined/acclimated for 1 week. The animals were housed in an environmentally controlled, air‐conditioned room maintained under specific pathogen‐free conditions with a 12‐h light–dark cycle (lighting from 7:00 a.m. to 7:00 p.m.) and at a temperature of 23 ± 1C°, relative humidity of 55 ± 5%, and a ventilation rate of about 15 times per hour. Animals had free access to a commercial pelleted diet (CRF‐1, Oriental Yeast Co., Ltd., Tokyo, Japan) and tap water ad libitum. All animal experiments were reviewed and approved by Institutional Animal Care and Use committee of the Toxicology Research Laboratories, Central Pharmaceutical Research Institute, Japan Tobacco Inc., and performed in accordance with Japanese Law for the Humane Treatment and Management of Animals (Law No. 105, as revised in 2013, issued on October 1, 1973).

### 2.2 Dosing of ETH

ETH (Sigma‐Aldrich Co. LLC., St. Louis, MO, USA), suspended in 0.5% methyl cellulose (MC) aqueous solution (Shin‐Etsu Chemical Co., Ltd., Tokyo, Japan), was administered orally at 0, 30, and 100 mg/kg once daily for 7 and 14 days to rats (eight animals per each sampling point at each dose level). All the animals were observed carefully for any clinical signs before and immediately after dosing. Body weights and food consumption were also recorded. On the day after the end of each dosing period, overnight‐fasted animals were anesthetized by isoflurane and then euthanized by exsanguination after blood sampling from the abdominal aorta. Blood samples treated with EDTA‐2K and heparin lithium as an anticoagulant were collected for lipidomics assays and clinical chemistry, respectively. The collected blood samples were centrifuged at 1750 × g for 30 min at room temperature or 4°C for EDTA‐2K or heparin lithium‐treated samples, respectively. The blood samples collected in nontreated tubes were allowed to stand at room temperature for at least 30 min and then centrifuged at approximately 1600 × g for 10 min at room temperature to separate the serum. Intact livers were removed from the bodies immediately after euthanization, weighed, and a part of the livers obtained from the left lateral lobe was frozen by liquid nitrogen and preserved in a deep freezer set at −80°C. The remaining liver was fixed by neutral buffered formalin for histopathological examinations.

### 2.3 Clinical chemistry

The plasma liver function‐related parameters (aspartate aminotransferase [AST], alanine aminotransferase [ALT], and glutamate dehydrogenase [GLDH]) and plasma lipids (TG, total cholesterol [T‐CH, and phospholipid [PL]) were measured by using an automated analyzer (TBA‐120FR, TOSHIBA Corporation, Tokyo, Japan) using standard reagents for clinical chemistry for AST, ALT, TG, T‐CH, and PL (Wako Pure Chemicals, Tokyo, Japan) and for GLDH (RANDOX Laboratories, Crumlin, UK).

### 2.4 Measurements of hepatic lipid contents

Approximately 200 mg liver samples were weighted in plastic tubes. Methanol (800 μl per 200 mg liver) and a zirconia ball (YTZ ball, φ 5 mm, NIKKATO Corporation, Osaka, Japan) were added to the samples and subsequently homogenized by using a mixer‐mill disruptor (TissueLyser, QIAGEN, Hilden, Germany). The homogenates (500 μl) and chloroform (1 ml) were mixed thoroughly by a vortex mixer. The mixtures were centrifuged at 1600 × g for 5 min. The supernatants were mixed with 0.5% sodium chloride aqueous solution (300 μl) and stirred thoroughly. The mixtures were centrifuged in the same conditions as described above. The chloroform phase was collected and evaporated to dryness with a centrifugal thickener (EZ‐2 PLUS, Genevac, Warminster, PA, USA). After evaporation, the residues were dissolved into 200 μl of isopropanol. The dissolved sample (50 μl) was stirred thoroughly with 4%bovine serum albumin aqueous solution (200 μl) and subsequently used for measuring the concentrations of TG, T‐CH, and PL by an automated analyzer by the enzymatic methods.

### 2.5 Histopathological examination of the liver

The liver was fixed in 10% phosphate‐buffered formalin, embedded in paraffin, sectioned at 4 μm, and stained with hematoxylin and eosin (H&E) and Oil Red O. Liver sections were examined microscopically by pathologists, and the pathological findings were recorded.

### 2.6 Lipidomics analysis

Plasma lipids were extracted from mixtures of 20 μl of plasma (EDTA‐2K) and 180 μl of methanol and isopropanol (1:1) containing 2 μM phosphatidylcholine (PC [12:0/12:0]; Avanti Polar Lipids, Alabaster, AL, USA) as an internal standard. Liver samples were thoroughly homogenized with methanol at the concentration of 20 mg/ml. Liver lipids were extracted by adding 100 μl of the homogenates with 100 μl of isopropanol containing 2 μM phosphatidylcholine (internal standard). The extracted lipids from plasma and liver were subsequently filtered through a FastRemover for Protein (0.20 μm) 96‐well (GL Science, Tokyo, Japan) using Microlab NIMBUS (Hamilton Robotics, Reno, NV, USA) to eliminate debris. The extracted samples were stored at the temperature of −80°C until use and were directly subjected to lipidomics. To obtain the lipidomics data, we performed reversed‐phase liquid chromatography (RPLC; Ultimate 3000, Thermo Fisher Scientific, Waltham, MA, USA) and a mass spectrometer (MS; Orbitrap Fusion, Thermo Fisher Scientific), as described previously (Saito et al., 2018; Saito, Ohno, & Saito, 2017). Compound Discoverer 2.1 (Thermo Fisher Scientific) was used with the raw data for peak extraction, annotation, identification, and lipid quantification, as described previously with a prior version of the software (Saito et al., 2018; Saito, Ohno, & Saito, 2017). For isomers (same class, carbon length, and number of double bonds) showing different retention times in RPLC, their name is added to an alphabet that discriminates the lipid from other isomers. The quantified raw data were normalized to the internal standard. The processed data for the lipid levels are presented in Supplementary Table S1 (plasma) and Supplementary Table S2 (liver).

### 2.7 Orthogonal partial least squares discriminant analysis (OPLS‐DA) and lipid identification

Lipidomics data from the plasma and liver of the control and ETH‐treated rats were loaded onto SIMCA‐P + 14 (Umetrics, Umea, Sweden), Pareto‐scaled, and analyzed using OPLS‐DA. Thereafter, the lipids with differences between the control rats and ETH‐treated rats were extracted. To sort these lipids, the value|p (corr)| > 0.8 in the loading s‐plot of the OPLS‐DA score, representing for the level of reliability, was adopted as a cutoff value. The composition of the sorted lipids was identified based on methods described previously (Ishikawa et al., 2014, 2016).

### 2.8 Serum TG‐rich lipoprotein profiling

Serum TG‐rich lipoprotein fractions were measured based on their particle size by the LipoSEARCH® method (Immuno‐Biological Laboratories Co., Ltd., Gunma, JAPAN) as reported by Toshima et al. (2013). This is briefly as follows: Lipoproteins were separated by permeation columns based on the size, and subsequently, TG concentrations were measured by an enzymatic reaction for TG. These sequential measurements were performed by using a gel‐permeation high‐performance liquid chromatography (GP‐HPLC). The chromatogram was analyzed by the Gaussian curve fitting method for the calculation of TG concentration in 4 or 20 lipoprotein fractions.

### 2.9 Gene expression profiling

The liver samples were homogenized by TissueLyser (QIAGEN), and total RNA was extracted by an RNeasy Mini Kit (QIAGEN). cDNA was synthesized from 2.0 μg extracted total RNA by using SperScript VILO Master Mix (Invitrogen, Carlsbad, CA, USA) and was diluted in five times the volume of Tris‐EDTA (TE) buffer (pH = 8.0, NIPPON GENE Co., LTD., Tokyo, Japan). cDNA solutions were subsequently diluted 10‐fold with MILLI‐Q water (Millipore Corporation, Darmstadt, Germany) and used for semiquantitative real‐time PCR. The mRNA levels were measured by a 7300 Real‐Time PCR System (Applied Biosystems, Waltham, MA, USA) using TaqMan Gene Expression Master Mix (Applied Biosystems). Data analysis was performed by SDS software (Applied Biosystems), and each of the mRNA expression levels was normalized based on the mRNA expression level of *β‐actin* (*Actb*). The mRNA expression levels of the following genes were measured: *fatty acid desaturase 1* (*Fads1*), *fatty acid desaturase 2* (*Fads2*), *glycerol‐3‐phosphate acyltransferase 1* (*Gpam*), and *lysophosphatidylcholine acyltransferase 3* (*Lpcat3*). The TaqMan probe mixtures described below were purchased from Applied Biosystems: *Actb* (Rn00667869_m1), *Fads1* (Rn00584915_m1), *Fads2* (Rn00580220_m1), *Gpam* (Rn00568620_m1), and *Lpcat3* (Rn01492616_m1).

### 2.10 Statistical analysis

All numerical data are shown as mean ± or + standard deviation. The differences in the data were analyzed using one‐way analysis of variance (ANOVA), followed by pairwise comparisons (Dunnett's test). The levels of significance were set at 5% and 1% (two‐tailed).

## 3 RESULTS

### 3.1 The relevancy of the dose levels of ETH between rats and humans

To confirm the relevancy of the dose levels of 30 and 100 mg/kg of ETH in rats, human equivalent dose (HED) was calculated based on body surface area using the following formulations (U.S. Food and Drug Administration, 2005):

$$\begin{array}{l} \text{Human equivalent dose}\left[\mathrm{mg}/\mathrm{kg}\right]\\ \;=\begin{array}{l} \text{Animal dose}\left[\mathrm{mg}/\mathrm{kg}\right]\times (\text{Animal weight}\left[\mathrm{kg}\right]\\ {÷\text{Human weight}\left[\mathrm{kg}\right])}^{0.33} \end{array}\\ \;=\text{Animal dose}\left[\mathrm{mg}/\mathrm{kg}\right]÷\text{Factor(for each animal)} \end{array}$$

By using a factor for the rat (= 6.2), the HED of ETH was calculated as 4.8 and 16.1 mg/kg for 30 and 100 mg/kg in rats. Because patients are prescribed ETH at 500 or 750 mg/day, the clinical dose level of ETH is calculated to be 10 or 15 mg/kg based on the body weight of a 50 kg/man (Chirehwa et al., 2021). Therefore, the dose levels of 30 and 100 mg/kg employed in the present study were considered to be close to the clinical dose.

### 3.2 Phenotype of the ETH‐induced hepatic steatosis

**FIGURE 1:**
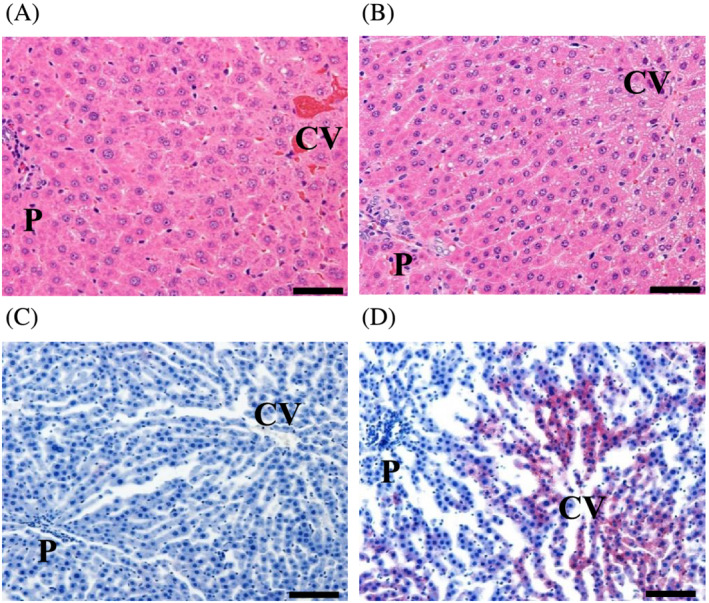
Histopathological fatty changes in the centrilobular hepatocytes induced by treatment with ETH. (A,B) H&E staining. (C,D) Oil Red O staining. (A,C) Control rats on Day 8. (B,D) ETH‐treated (100 mg/kg) rats on Day 8. Scale bar = 50 μm; CV, central vein; P, portal area. Representative findings for fatty change of hepatocytes are shown

There were no noteworthy findings in the clinical observations or body weights throughout the dosing period at either dose level, whereas food consumption was slightly decreased in the first week in the animals at 100 mg/kg of ETH (Table 1). Liver weights relative to body weights were increased in the animals treated at 100 mg/kg of ETH on Day 15 (after 14 days dosing) (Table 1). Hepatic TG contents tended to be increased in the animals treated at 100 mg/kg of ETH on Days 8 (after 7 days dosing) and 15. Hepatic T‐CH contents were significantly increased at both dose levels of ETH on Day 8 and at 100 mg/kg of ETH on Day 15. There were no changes in hepatic PL contents at either dose level. In the clinical chemistry, plasma GLDH levels were significantly increased at 100 mg/kg of ETH on Day 15. There were no changes in plasma AST or ALT levels at either dose level on both Days 8 and 15. Plasma TG levels tended to be decreased at both dose levels of ETH on Day 8 and at 30 mg/kg of ETH on Day 15 and were significantly decreased at 100 mg/kg of ETH on Day 15. Plasma T‐CH levels tended to be decreased at 100 mg/kg of ETH on Day 15. Plasma PL levels were significantly decreased at 100 mg/kg of ETH on Day 15. At necropsy, pale discoloration of the liver was observed macroscopically in one or all the animals treated at 100 mg/kg of ETH on Day 8 or 15, respectively (Table 2). In the histopathological examination of the liver, vacuolation of the centrilobular hepatocytes was observed at 100 mg/kg of ETH on Day 8 and at both dose levels of ETH on Day 15 (Figure 1 and Table 2). The vacuolation stained positively with Oil Red O (Figure 1), indicating that the vacuoles were contained neutral lipids.

**TABLE 1:** Summary of the parameters in the control and ETH‐treated rats

	Sampling point^a^	Control	ETH 30 mg/kg	ETH 100 mg/kg
Final body weight (g)	Day 8 Day 15	238.68 ± 13.53 302.30 ± 18.11	234.04 ± 10.81 300.06 ± 11.33	236.54 ± 13.37 288.74 ± 14.58
Food consumption (g/day)	Days 5–7 Days 10–14	27.78 ± 1.98 29.98 ± 2.13	26.24 ± 1.73 28.79 ± 1.84	24.69 ± 2.03** 27.93 ± 2.34
AST (U/dL)	Day 8 Day 15	74.1 ± 5.6 75.0 ± 5.7	69.6 ± 6.2 73.8 ± 8.9	69.5 ± 6.7 76.6 ± 6.2
ALT (U/dL)	Day 8 Day 15	29.1 ± 3.7 29.3 ± 4.3	27.3 ± 2.6 31.0 ± 4.1	29.8 ± 6.4 33.1 ± 2.9
GLDH (U/dL)	Day 8 Day 15	33.4 ± 9.8 31.6 ± 5.3	23.9 ± 4.4 28.5 ± 7.4	35.0 ± 14.2 51.4 ± 18.1**
TG (mg/dL)	Day 8 Day 15	42.0 ± 16.9 53.3 ± 12.4	30.0 ± 7.1 53.3 ± 12.4	30.3 ± 8.0 29.3 ± 6.0**
T‐CH (mg/dL)	Day 8 Day 15	57.4 ± 10.3 55.6 ± 10.1	50.0 ± 7.3 55.0 ± 15.0	48.5 ± 11.3 43.6 ± 7.9
PL (mg/dL)	Day 8 Day 15	89.9 ± 12.4 89.6 ± 9.1	79.6 ± 6.6 86.9 ± 16.0	78.3 ± 11.2 73.9 ± 10.2*
Relative liver weight (g/100 g body weight)	Day 8 Day 15	3.283 ± 0.189 3.065 ± 0.200	3.225 ± 0.141 3.013 ± 0.126	3.435 ± 0.194 3.336 ± 0.159**
Hepatic TG level (mg/g liver)	Day 8 Day 15	6.78 ± 2.68 14.28 ± 7.40	6.75 ± 3.56 14.20 ± 3.98	10.40 ± 6.07 19.03 ± 6.20
Hepatic T‐CH level (mg/g liver)	Day 8 Day 15	2.63 ± 0.43 3.10 ± 0.34	3.33 ± 0.67* 3.30 ± 0.60	3.60 ± 0.52** 4.05 ± 0.75**
Hepatic PL level (mg/g liver)	Day 8 Day 15	16.28 ± 1.36 18.35 ± 1.08	16.48 ± 0.81 19.10 ± 2.50	16.53 ± 1.45 17.65 ± 0.96

*Note*: Eight animals were assessed in each sampling point at each dose level.

Abbreviations: ALT, alanine aminotransferase; AST, aspartate aminotransferase; ETH, ethionamide; GLDH, glutamate dehydrogenase; PL, phospholipids; T‐CH, total cholesterols; TG, triglycerides.

^a^ Days 8 and 15 mean the day after 7 and 14 days of dosing, respectively.

* *P* < 0.05, significantly different from control (Dunnett's test).

^**^ *P* < 0.01, significantly different from control (Dunnett's test).

**TABLE 2:** Summary of necropsy and histopathology of the liver

	ETH 0 mg/kg		ETH 30 mg/kg		ETH 100 mg/kg	
Sampling point^a^	Day 8	Day 15	Day 8	Day 15	Day 8	Day 15
Number of animals	8	8	8	8	8	8
Macroscopic findings^b^						
Discoloration, pale	−: 8	−: 8	−: 8	−: 8	−: 7, P: 1	P: 8
Microscopic findings^c^						
Vacuolation, hepatocyte, centrilobular	−: 8	−: 8	−: 8	−: 4, ±: 2, +: 2	±: 6, +: 1, 2+: 1	±: 2, +: 4, 2+: 2

^a^ Days 8 and 15 mean the day after 7 and 14 days of dosing, respectively.

^b^ Criteria for grading macroscopic findings: −, no abnormal changes; P, presence of the findings. Number of animals in which the grade was observed.

^c^ Criteria for grading microscopic findings: −, no abnormal changes; ±, very slight; +, slight; 2+, moderate; 3+, severe. Number of animals in which the grade was observed.

### 3.3 Lipidomics analysis for the plasma and liver

**FIGURE 2:**
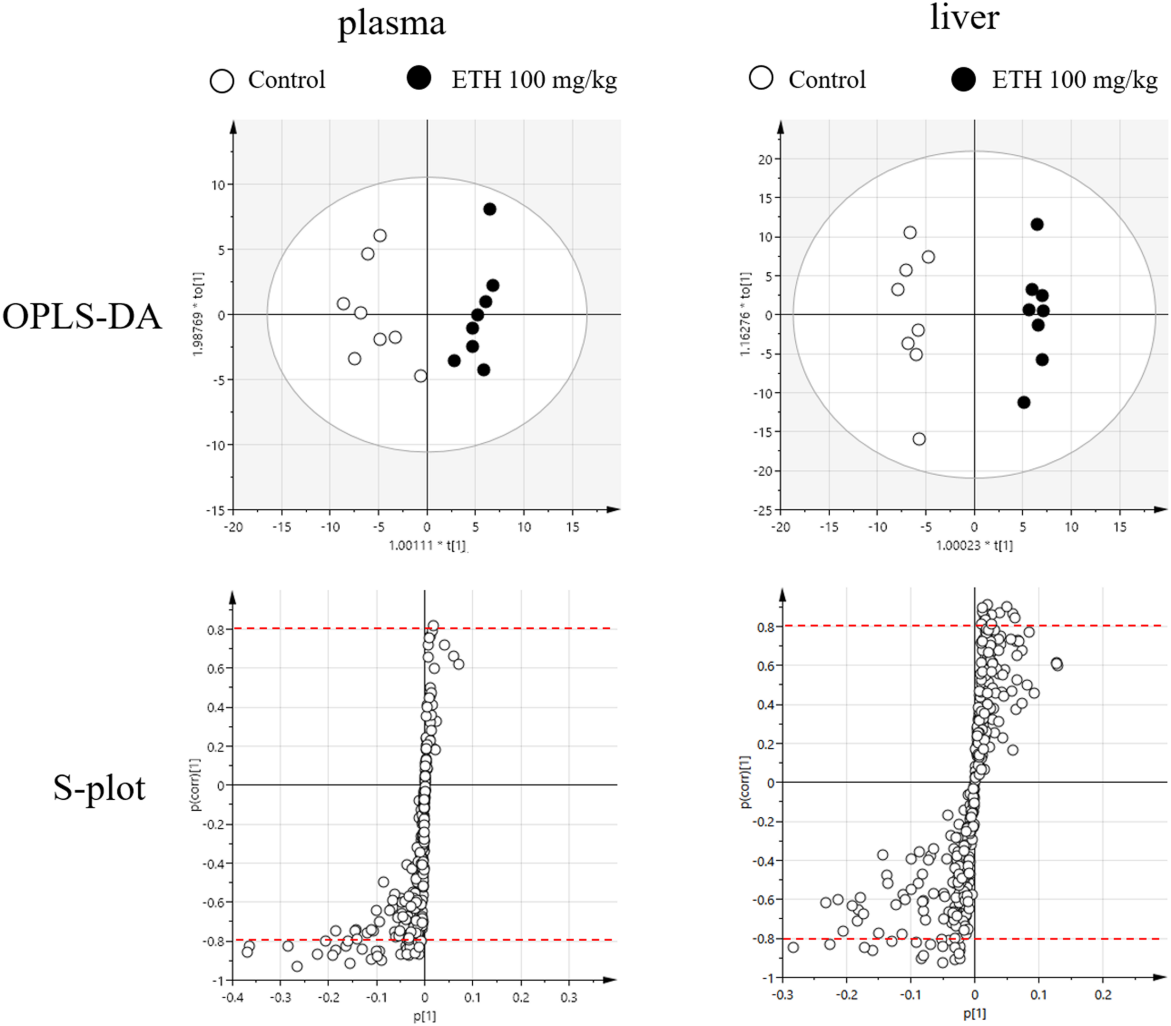
OPLS‐DA and s‐plot of lipidomics data from plasma (left) and liver (right). OPLS‐DA score plot and loading s‐plot were described by using data obtained from LC/MS analysis. Each dot in OPLS‐DA score plot represents each animals of control (white) and ETH‐treated rats (black). Each dot in loading s‐plot represents each measured lipid in the lipidomics analysis. The thresholds are denoted by dotted red lines (|p (corr)| = 0.8)

To investigate the biomarkers for ETH‐induced hepatic steatosis, lipidomics analysis was conducted using plasma and liver samples from rats treated with ETH for 14 days at 0 and 100 mg/kg. Three hundred and nineteen lipids from the plasma and 349 lipids from the liver were measured. The measurement data of the lipids were subsequently loaded into OPLS‐DA analysis, and the lipid alterations were identified by calculating the loading s‐plot scores. Fine discrimination between the control and ETH‐treated samples was obtained in OPLS‐DA plots of both the plasma and liver lipidomics data (Figure 2). Based on the threshold values (|p (corr)| > 0.8) in the s‐plot, 35 lipids each from the plasma and liver were identified as distinctive lipids, which discriminated between the control and ETH‐treated rats (Tables 3 and 4). In the plasma, ceramide (Cer), lysophosphatidylcholine (LPC), phosphatidylcholine (PC), sphingomyelin (SM), diacylglycerol (DG), and TG were decreased in the ETH‐treated rats (Table 3). On the other hand, in the liver, LPC, lysophosphatidylethanolamine (LPE), PC, phosphatidylethanolamine (PE), ether‐phosphatidylethanolamine (PEe), phosphatidylinositol (PI), and SM were mainly decreased, and TGs were increased in the ETH‐treated rats (Table 4). Structural analyses of the lipids showed that altered TGs in the plasma and liver were highly containing saturated fatty acids (e.g., palmitic acid [16:0] and stearic acid [18:0]).

**TABLE 3:** Characterized plasma lipids altered by ETH treatment (100 mg/kg, Day 15) in OPLS‐DA

Class	Name	Confirmed structure	RT	Response by ETH
Cer	Cer(d40:1)	Not confirmed	17.043	Decreased
LPC	LPC(18:0)a	LPC(18:0)	5.731	Decreased
LPC	LPC(18:0)b	LPC(18:0)	6.036	Decreased
PC	PC(37:4)	PC(17:0/20:4)	12.778	Decreased
PC	PC(38:4)	PC(18:0/20:4)	13.528	Decreased
PC	PC(38:5)	PC(18:0/20:5)	12.57	Decreased
PC	PC(39:4)	PC(19:0/20:4)	14.062	Decreased
PC	PC(40:4)	Not confirmed	14.914	Decreased
PC	PC(40:8)	PC(20:4/20:4)	10.617	Decreased
PI	PI(40:5)	Not confirmed	12.308	Increased
SM	SM(d41:2)	Not confirmed	15.669	Decreased
DG	DG(36:4)	DG(18:2/18:2)	14.351	Decreased
DG	DG(36:5)	Not confirmed	13.306	Decreased
TG	TG(51:3)	TG(15:0/18:1/18:2)	19.54	Decreased
TG	TG(51:4)	TG(15:0/18:2/18:2)	19.194	Decreased
TG	TG(52:3)	TG(16:0/18:1/18:2)	19.733	Decreased
TG	TG(52:4)	TG(16:0/18:2/18:2)	19.402	Decreased
TG	TG(52:5)	TG(16:0/18:2/18:3)	19.089	Decreased
TG	TG(53:4)	TG(17:1/18:1/18:2), TG(17:0/18:2/18:2)	19.571	Decreased
TG	TG(53:6)	TG(15:0/18:2/20:4)	19.063	Decreased
TG	TG(54:4)	TG(18:1/18:1/18:2)	19.741	Decreased
TG	TG(54:5)	TG(18:1/18:2/18:2)	19.403	Decreased
TG	TG(54:6)a	Not confirmed	19.066	Decreased
TG	TG(54:6)b	TG(16:0/18:2/20:4)	19.267	Decreased
TG	TG(55:6)	TG(17:0/18:2/20:4), TG(16:0/18:2/21:4)	19.427	Decreased
TG	TG(56:6)	TG(18:0/18:2/20:4)	19.603	Decreased
TG	TG(56:8)	TG(16:0/18:2/22:6), TG(18:2/18:2/20:4)	19.087	Decreased
TG	TG(56:9)	TG(18:2/18:2/20:5), TG(18:2/18:3/20:4)	18.583	Decreased
TG	TG(56:10)	TG(18:2/18:3/20:5)	18.204	Decreased
TG	TG(57:8)	TG(17:0/−/22:6)	19.275	Decreased
TG	TG(57:9)a	Not confirmed	18.717	Decreased
TG	TG(57:9)b	TG(17:1/18:2/22:6)	18.89	Decreased
TG	TG(58:8)	TG(18:0/18:2/22:6), TG(18:0/20:4/20:4)	19.458	Decreased
TG	TG(58:9)	TG(18:1/18:2/22:6)	19.092	Decreased
TG	TG(58:10)	TG(18:2/18:2/22:6), TG(18:2/20:4/20:4)	18.719	Decreased

*Notes*: The number in parentheses represents the number of carbons and degree of unsaturated, respectively. When more than two lipid molecules were identified as isomers that have same lipids class, carbon length, and number of double bonds, each lipid molecule is described alphabetically to distinguish them.

Abbreviations: Cer, ceramide; DG, diacylglycerol; LPC, lysophosphatidylcholine; PC, phosphatidylcholine; PI, phosphatidylinositol; RT, retention time (min); SM, sphingomyelin; TG, triglyceride.

**TABLE 4:** Characterized liver lipids altered by ETH treatment (100 mg/kg, Day 15) in OPLS‐DA

Class	Name	Confirmed structure	RT	Response by ETH
Cer	Cer(d41:1)	Cer(d18:1/23:0)	17.413	Increased
LPC	LPC(18:0)	LPC(18:0)	6.02	Decreased
LPE	LPE(18:0)	LPE(18:0)	6.085	Decreased
PC	PC(36:6)	PC(14:0/22:6)	10.106	Increased
PC	PC(37:2)	Not confirmed	14.53	Decreased
PC	PC(38:4)	PC(18:0/20:4)	13.503	Decreased
PC	PC(39:4)	PC(19:0/20:4)	14.267	Decreased
PC	PC(40:4)	Not confirmed	14.9	Decreased
PE	PE(34:2)	PE(16:0/18:2)	12.622	Decreased
PE	PE(36:1)	PE(18:0/18:1)	15.14	Increased
PE	PE(36:4)	PE(16:0/20:4)	12.316	Decreased
PE	PE(36:5)	PE(16:0/20:5)	11.301	Decreased
PE	PE(37:4)	PE(17:0/20:4)	13.112	Decreased
PE	PE(38:4)	PE(18:0/20:4)	13.908	Decreased
PE	PE(38:5)	PE(18:0/20:5), PE(16:0/22:5)	12.863	Decreased
PE	PE(39:4)a	PE(19:0/20:4)	14.358	Decreased
PE	PE(39:4)b	PE(19:0/20:4)	14.581	Decreased
PE	PE(40:8)	PE(18:2/22:6), PE(20:4/20:4)	10.843	Increased
PEe	PE(38:5e)	PE(18:1e/20:4)	14.562	Decreased
PEe	PE(40:6e)	PE(18:1e/22:5)	14.608	Decreased
PI	PI(37:4)	PI(−/20:4)	11.435	Decreased
PS	PS(38:4)	PS(18:0/20:4)	12.485	Decreased
PS	PS(38:6)	PS(16:0/22:6)	10.59	Increased
SM	SM(d34:1)	SM(d34:1)	11.701	Decreased
SM	SM(d40:2)	Not confirmed	15.048	Decreased
SM	SM(d41:2)	Not confirmed	15.633	Decreased
TG	TG(52:1)	TG(16:0/18:0/18:1)	20.399	Increased
TG	TG(54:1)	TG(18:0/18:0/18:1)	20.699	Increased
TG	TG(54:2)	TG(18:0/18:1/18:1), TG(16:0/18:1/20:1)	20.392	Increased
TG	TG(55:2)	Not confirmed	20.531	Increased
TG	TG(56:2)	TG(18:0/18:1/20:1), TG(16:0/18:1/22:1)	20.684	Increased
TG	TG(58:2)	Not confirmed	20.963	Increased
TG	TG(58:3)	Not confirmed	20.693	Increased
TG	TG(60:3)	Not confirmed	20.983	Increased
TG	TG(64:15)	Not confirmed	18.42	Increased

*Notes*: The number in parentheses represents the number of carbons and degree of unsaturated, respectively. When more than two lipid molecules were identified as isomers that have same lipids class, carbon length, and number of double bonds, each lipid molecule is described alphabetically to distinguish them.

Abbreviations: Cer, ceramide; DG, diacylglycerol; LPC, lysophosphatidylcholine; LPE, lysophosphatidylethanolamine; PC, phosphatidylcholine; PE, phosphatidylethanolamine; PEe, ether‐phosphatidylethanolamine; PI, phosphatidylinositol; PS, phosphatidylserine; RT, retention time (min); SM, sphingomyelin; TG, triglyceride.

### 3.4 Relationships of the altered lipids between the plasma and the liver

**FIGURE 3:**
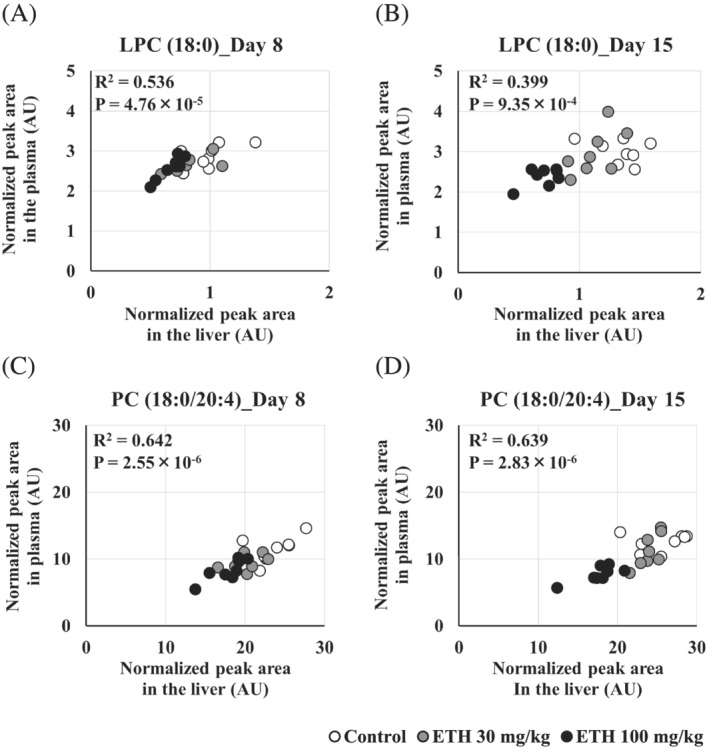
Correlation of LPC (18:0) and PC (18:0/20:4) in the plasma and liver. Each plot represents data from each individual animal. (A) LPC (18:0) on Day 8, (B) LPC (18:0) on Day 15, (C) PC (18:0/20:4) on Day 8, D: PC (18:0/20:4) on Day 15. Days 8 and 15 mean the sampling points. Goodness‐of‐fit (R^2^) and *P*‐value of regression analysis for each lipid were calculated with all data points. AU, arbitrary unit

**FIGURE 4:**
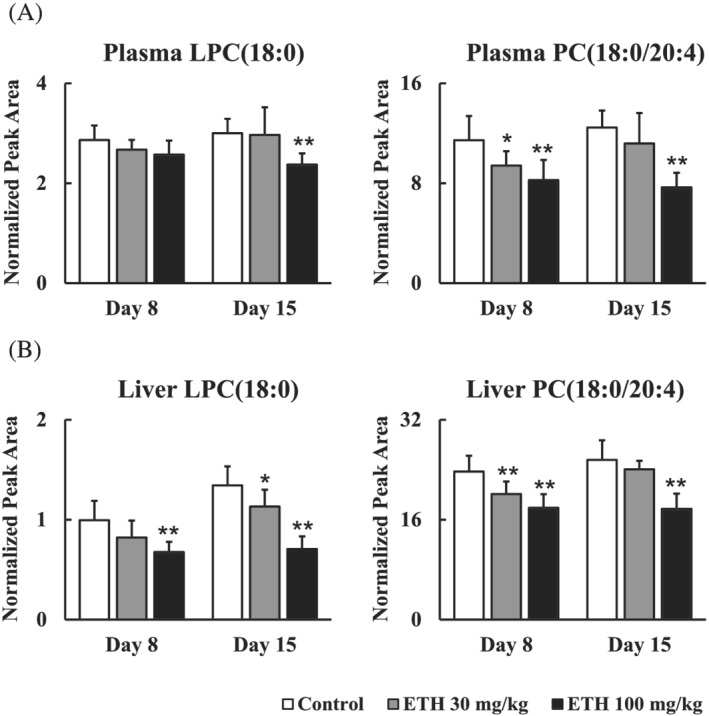
Time‐ and dose‐dependent changes in the levels of LPC (18:0) and PC (18:0/20:4). (A) LPC (18:0) level in the plasma, (B) PC (18:0/20:4) level in the plasma, (C) LPC (18:0) level in the liver, (D) PC (18:0/20:4) level in the liver. Days 8 and 15 mean the sampling points. Each bar represents mean + S.D. with each sample (*n* = 8). Significantly different from control (Dunnett's test): ^*^ *P* < 0.05, ^**^ *P* < 0.01

To investigate the biomarkers that reflected the changes for the ETH‐induced hepatic steatosis, we extracted the lipids altered in both plasma and liver. As the result, LPC (18:0) and PC (38:4) were identified as lipids that decreased in both plasma and liver. From structural analyses, PC (38:4) was identified as PC (18:0/20:4), containing stearic acid (18:0) and arachidonic acid (20:4). These lipids were highly positively correlated between the plasma and liver (Figure 3A,B). Both LPC (18:0) and PC (18:0/20:4) decreased dose‐dependently on Days 8 and 15 (Figure 4A,B).

### 3.5 Lipoprotein profiling in the serum

**FIGURE 5:**
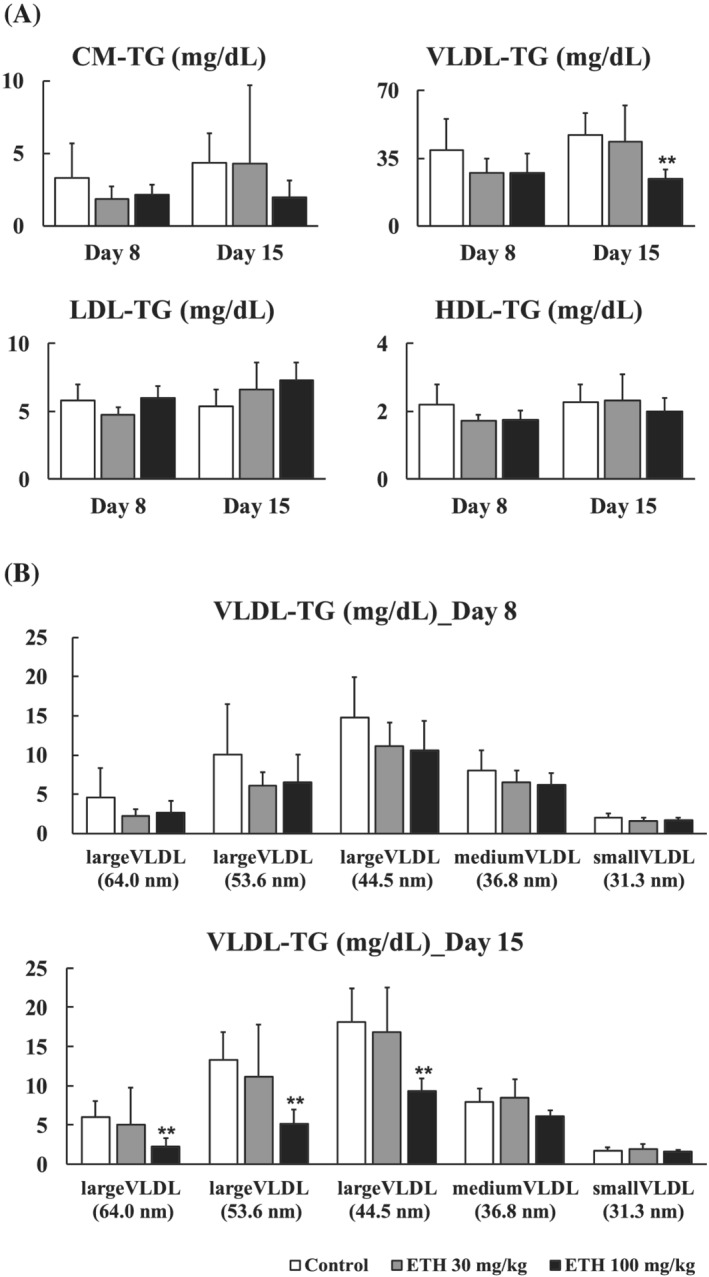
Changes in TG levels in the serum lipoprotein fractions. (A) Serum TG levels in four lipoprotein fractions. (B) Serum TG levels in detailed VLDL fractions. The number in parentheses means diameter of lipoprotein. CM, chylomicron; HDL, high‐density lipoprotein; LDL, low‐density lipoprotein; VLDL, very low‐density lipoprotein. Each bar represents mean + SD with each sample (*n* = 8). Significantly different from control (Dunnett's test): ^*^ *P* < 0.05, ^**^ *P* < 0.01

The PCs containing arachidonic acids (e.g., PC [18:0/20:4]) were reported to be involved in the excretion of TG‐rich very low‐density lipoprotein (VLDL) into the blood from the liver (Rong et al., 2015). Because we revealed that ETH decreased PC containing arachidonic acid in the lipidomics study, we investigated the effect of ETH treatment on the TG concentration in the serum VLDL. Serum TG‐rich VLDL fraction tended to be decreased at both dose levels of ETH on Day 8 and was significantly decreased at 100 mg/kg of ETH on Day 15, but not in chylomicron (CM), low‐density lipoprotein (LDL), and high‐density lipoprotein (HDL) fractions (Figure 5A). Moreover, in the 20‐subclass profiling of the TG‐rich lipoproteins based on the particle size of the lipoproteins, the large‐sized VLDL‐TG tended to be decreased at both dose levels of ETH on Day 8 and significantly decreased on Day 15, but the degree of the decrease tended to diminish with decreased particle size of the VLDL (Figure 5B). Similar trends were observed in plasma T‐CH levels in the lipoprotein fractions (Supplementary Figure S1A,B).

### 3.6 Gene expression profiling related to elongation and desaturation steps of the fatty acids

**FIGURE 6:**
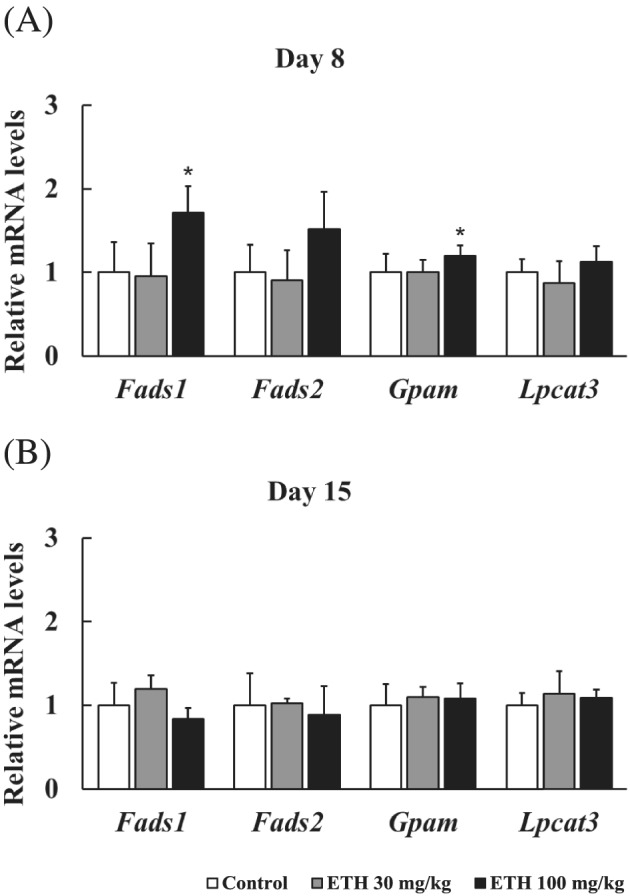
Hepatic relative mRNA levels of *Fads1*, *Fads2*, *Gpam*, and *Lpcat3* genes. *Fads1*, *fatty acid desaturase 1*; *Fads2*, *fatty acid desaturase 2*; *Gpam*, *glycerol‐3‐phosphate acyltransferase 1, mitochondrial*; *Lpcat3*, *lysophosphatidylcholine acyltransferase 3*. Days 8 and 15 mean the sampling points. Each bar represents mean + SD with each sample (*n* = 5). Significantly different from control (Dunnett's test): ^*^ *P* < 0.05

Hepatic mRNA expression levels of *Fads1*, *Fads2*, *Gpam*, and *Lpcat3* were measured as genes related to the synthesis of PCs containing arachidonic acid. The *Fads1* and *Fads2* are related to long‐chain fatty acid metabolism (Glaser et al., 2010). The *Gpam* and *Lpcat3* are enzymes involved in the first step of glycerolipid synthesis and the metabolism of lysophosphatidylcholine in Lands cycle, respectively, and they influence the synthesis of PCs containing arachidonic acid (Hammond et al., 2002; Rong et al., 2015). *Fads1* mRNA expression levels were significantly increased at 100 mg/kg of ETH on Day 8 (Figure 6). Moreover, *Fads2* mRNA expression levels tended to be increased at 100 mg/kg of ETH on Day 8. *Gpam* mRNA expression levels were significantly increased at 100 mg/kg of ETH on Day 8. On the other hand, *Lpcat3* mRNA expression levels did not change on either day.

## 4 DISCUSSION

Drug‐induced hepatic steatosis (DIS) is induced by some DILI‐inducing drugs such as zidovudine, methotrexate, and valproic acid in rats and humans (Banerjee et al., 2013; Custer et al., 1977; Kesterson et al., 1984). Moreover, some drugs are known to cause steatohepatitis via cell stress such as impairment of mitochondrial function followed by production of reactive oxygen species (Begriche et al., 2011; Letteron et al., 1996). From these reports, DIS is considered to be one of the causes and risk factors for DILI and clarifying the developmental mechanisms of DIS is important to understand the pathology of, and to predict, the onset of DILI. The pathogenesis of DIS is associated with several mechanisms including promotion of fatty acid synthesis, deterioration of fatty acid oxidation via mitochondrial impairment, and suppression of TG release from the hepatocytes. ETH is a drug for the treatment of multiresistant tuberculosis (Tiberi et al., 2019) and is known to cause DIS (Hollinrake, 1968; Lees, 1963; Moulding & Goldstein, 1962; Phillips & Tashman, 1963; Sahini et al., 2014).

In the present study, to identify biomarkers for ETH‐induced steatosis, we produced a drug‐induced steatosis model by oral administration of ETH to rats. Based on the histopathological alterations and changes in hepatic TG and T‐CH contents, hepatic steatosis was considered to be induced within a 1‐week treatment of ETH in rats without inducing necrotic changes or increases in liver function tests including plasma transaminase levels (Figure 1 and Tables 1 and 2). These results were considered to be appropriate for the investigation of alterations of lipid profile in hepatic steatosis and biomarkers before induction of liver injury by ETH.

Lipidomics analysis clarified that PC (18:0/20:4) whose fatty acids are stearic acid (18:0) and arachidonic acid (20:4) were decreased before the onset of hepatic steatosis (Figures 3 and 4). These results indicated a potential of PC (18:0/20:4) as one of the biomarkers for the ETH‐induced hepatic steatosis. Many studies have clarified that PC levels in the liver or plasma are altered in patients with hepatic steatosis. In the nonalcoholic fatty liver disease (NAFLD), hepatic levels of total PC and PCs containing arachidonic acid were decreased compared with those in healthy subjects (Puri et al., 2007). Fatty acid profiles in the plasma PC were also altered in NAFLD patients, increases in PCs containing palmitoleic acid or stearic acid, for instance (Puri et al., 2009). Patients with hereditary hemochromatosis that shows hepatic steatosis had significantly different serum PC profiles compared with healthy subjects (e.g., increases in PCs containing polyunsaturated fatty acid) (Seeßle et al., 2020). Based on these reports, the plasma PC profiles might characterize various types of hepatic steatosis. Furthermore, decreases in the hepatic and plasma PCs containing arachidonic acid were also noted in tamoxifen‐induced hepatic phospholipidosis in rats (Saito, Goda, et al., 2017). Hence, the levels of PCs containing arachidonic acid are considered to be broadly altered in drug‐induced hepatic steatosis and phospholipidosis.

PC is mainly synthesized in the liver and subsequently secreted into the blood as one of the components of VLDL (Law et al., 2019). Two pathways involved in PC synthesis are known: the Kennedy pathway and the PE methyltransferase (PEMT) pathway (Jacobs et al., 2004). The former is a main pathway in which PC is synthesized from cytidine diphosphate (CDP)–choline, and the latter is a minor pathway in which PC is synthesized by continuous methylation of PE by PEMT (Cole et al., 2012; Jacobs et al., 2004). The synthesized PC is hydrolyzed at the *sn‐2* fatty acid chain by phospholipase A2 in the blood, resulting in the formation of LPC. The LPC is transported into the liver from the blood and acylated at the *sn‐2* position by lysophosphatidylcholine acyltransferase (LPCAT), an enzyme located in the liver, resulting in the reformation of PC. These sequential metabolism pathways of PC and LPC are referred as Lands cycle (Law et al., 2019). Rong et al. (2015) reported that *Lpcat3* knockout mice showed hepatic steatosis with the decreased content of PCs containing arachidonic acid in the liver. In this knockout mouse, the membrane fluidity of the hepatocytes is decreased, leading to suppression of VLDL excretion from the liver. Similar examples have been also reported for relationships between decreases in PC synthesis and VLDL excretion (Jacobs et al., 2004; Yao & Vance, 1988). Based on these reports, we hypothesize that the decrease in PCs containing arachidonic acid in the liver causes suppression of TG‐rich VLDL excretion from the liver in rats treated with ETH. In the present study, we measured serum TG and T‐CH levels in the lipoprotein fractions and found as expected that the decreases in serum TG and T‐CH levels were related to a decrease in those in VLDL, especially large‐sized VLDL, but not in CM, LDL, or HDL (Figure 5). Nascent VLDL is large‐sized VLDL and is excreted from the liver. Immediately after excretion, the large‐sized VLDL is metabolized into intermediate‐density lipoprotein (IDL) by lipoprotein lipase (LPL) and subsequently metabolized to LDL by lipase (Holmes & Ala‐Korpela, 2019). Our results indicated that ETH suppressed excretion of TG‐ and T‐CH‐rich VLDL from the liver. This is considered to be one of the major mechanisms in ETH‐induced hepatic steatosis.

PCs synthesized *de novo* usually have oleic acid at the *sn‐2* position, and PCs containing arachidonic acid are synthesized by Lands cycle where PCs are hydrolyzed at the *sn‐2* position and subsequently acylated with arachidonic acid, referred to as the fatty acid remodeling systems (Yamashita et al., 2017). Based on this report, synthesis of arachidonic acid (20:4) or fatty acid remodeling system was assumed to be related to the mechanism of decreases in PC (18:0/20:4) levels in ETH‐treated rats. However, increases in hepatic mRNA expression levels of *Fads1* and *Fads2*, genes related to the arachidonic acid (20:4) synthesis, indicated that synthesis of arachidonic acid was not impaired (Figure 6A). Moreover, the lack of changes in expression levels of *Lpcat3*, a fatty acid remodeling enzyme, implied no effects of ETH on the remodeling systems (Figure 6A). Increases in the expression levels of *Gpam*, a rate‐limiting enzyme for TG and PL synthesis, suggested that the syntheses of TG and PL were promoted by ETH treatment (Figure 6A). The PCs containing arachidonic acid at the *sn‐2* position are known to decrease in *Gpam*‐overexpressed mice and to increase in *Gpam* knockout mice (Hammond et al., 2002; Lindén et al., 2006). This knowledge from the previous reports is supportive to our results that the ETH‐treated rats had decreased PC (18:0/20:4) accompanied by increased *Gpam* expression levels. Taken together, neither decrease in arachidonic acid synthesis nor fatty acid remodeling is involved in the decrease in PC (18:0/20:4). PCs are synthesized from DG and CDP‐choline in the Kennedy pathway (Cole et al., 2012). Therefore, the remaining possibility of decrease in PC (18:0/20:4) levels in the ETH‐treated rats is considered to be related to choline utilization during PC biosynthesis, though further study is needed to investigate the precise mechanism.

Free fatty acids have the potential to induce cell toxicity in hepatocytes (Malhi et al., 2006). The toxic effect differs in each fatty acid species: saturated fatty acids (e.g., palmitic acid [16:0] and stearic acid [18:0]) are more toxic, whereas unsaturated fatty acids (e.g., palmitoleic acid [16:1] and oleic acid [18:1]) are less toxic (Malhi et al., 2006). Free saturated fatty acids are known to inhibit the synthesis of TG, however, they are incorporated into TG in the presence of unsaturated fatty acids (Listenberger et al., 2003; Mantzaris et al., 2011). Mantzaris et al. (2011) reported that saturated free fatty acid induced cell toxicity via ER stress, but the toxic effect was attenuated through the promotion of free fatty acids incorporation into TG in the presence of unsaturated fatty acids. ETH is also reported to induce ER stress in rats (Sutherland et al., 2018) and to cause hepatic necrosis at 300 mg/kg in rats from Day 4, by reference to the Open TG‐GATEs database (Igarashi et al., 2015). In the multivariate analysis conducted in the present study, ETH increased hepatic TG species in rats, especially TGs highly containing saturated fatty acids (i.e., a low degree of unsaturated fatty acids) (Table 4). The increase in TGs containing saturated fatty acids in the liver of the ETH‐treated rats might be adaptive changes against hepatocyte toxicity caused by saturated fatty acids.

**FIGURE 7:**
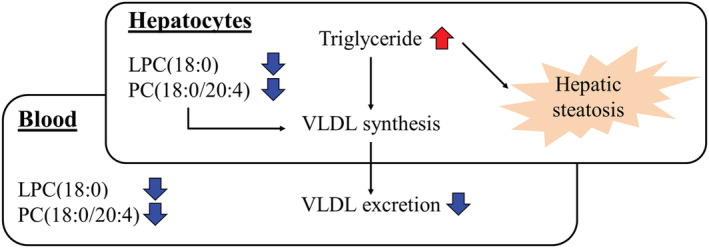
ETH decreased the levels of LPC (18:0) and PC (18:0/20:4) and showed hepatic steatosis in rats. TG‐rich VLDL excretion where PC containing arachidonic acid involved were decreased by treatment with ETH in rats. These molecular biological changes were considered to be related to ETH‐induced hepatic steatosis. Red arrow means “increase,” and blue arrow means “decrease” by ETH treatment [Colour figure can be viewed at wileyonlinelibrary.com]

In conclusion, the present study showed the mechanistic correlation between plasma PC (18:0/20:4) and the hepatic steatosis followed by a decrease in TG‐rich VLDL secretion from the hepatocytes in ETH‐treated rats (Figure 7). The mechanism of hepatic steatosis might capture the early‐phase in the pathogenesis of the liver injury caused by ETH. Although further studies are required, the present study showed a potential of PC (18:0/20:4) as one of the biomarkers for ETH‐induced hepatic steatosis.

## CONFLICT OF INTEREST

The authors did not report any conflict of interest.

## Supporting information




**Supplementary Figure S1** Changes in T‐CH levels in the serum lipoprotein fractions. A: Serum T‐CH levels in four lipoprotein fractions. B: Serum T‐CH levels in detailed VLDL fractions. The number in parentheses means diameter of lipoprotein. CM: Chylomicron, VLDL: Very Low‐Density Lipoprotein, LDL: Low‐Density Lipoprotein, HDL: High‐Density Lipoprotein. Each bar represents mean + S.D. with each sample (n = 8). Significantly different from control (Dunnett test): * P < 0.05, ** P < 0.01.

**Supplementary Table S1** Normalized levels of extracted lipids from rat plasma
**Supplementary Table S2** Normalized levels of extracted lipids from rat liver

## Data Availability

The data that supports the findings of this study are available in the supplementary material of this article
